# Resilience in Parents of Childhood Cancer Survivors: Results From the Swiss Childhood Cancer Survivors Study—Parents

**DOI:** 10.1002/pon.70443

**Published:** 2026-03-30

**Authors:** Peter Francis Raguindin, Anica Ilic, Julia Baenziger, Marc Ansari, Mutlu Kartal‐Kaess, Katharina Roser, Gisela Michel

**Affiliations:** ^1^ Faculty of Health Sciences and Medicine University of Lucerne Lucerne Switzerland; ^2^ Department of Behavioural Medicine Institute of Basic Medical Sciences Faculty of Medicine University of Oslo Oslo Norway; ^3^ Division of Pediatric Oncology and Hematology, Department of Women, Child and Adolescent University Geneva Hospitals Geneva Switzerland; ^4^ Faculty of Medicine, Department of Pediatrics, Gynecology and Obstetrics CANSEARCH Research Platform for Pediatric Oncology and Hematology University of Geneva Geneva Switzerland; ^5^ Division of Pediatric Hematology & Oncology, Department of Pediatrics Inselspital ‐ University Hospital Bern University of Bern Bern Switzerland; ^6^ Department for BioMedical Research University of Bern Bern Switzerland

**Keywords:** childhood cancer, parents, psychosocial outcomes, resilience, survivorship

## Abstract

**Background:**

Resilience, or the ability to adapt, recover, and thrive under stress, is crucial for mental health, but remains understudied in parents of childhood cancer survivors (CCS‐parents). We aimed to: (a) describe the resilience of CCS‐parents, comparison parents (parents from general population), and the Swiss general population (SGP), (b) compare resilience of CCS‐parents and comparison‐parents, and (c) identify characteristics associated with resilience in CCS‐parents.

**Methods:**

This is a cross‐sectional population‐based study including CCS‐parents who were identified through the Swiss Childhood Cancer Registry, and comparison‐parents from a representative sample of the general population. Resilience was measured using the Connor‐Davidson Resilience Scale (CD‐RISC). Linear regression was used to estimate differences between CCS‐parents and comparison parents, adjusting for confounders. Characteristics associated with resilience in CCS‐parents were identified through multilevel linear regression with parent couples clustered at the family level.

**Results:**

Data from 468 CCS‐parents (mean age = 62.2 years; 58.5% female), 473 comparison parents (mean age = 62.1 years; 57.5% female) and 1246 individuals from the SGP (mean age = 48.9 years; 58.2% female) were analyzed. Mean resilience in the SGP was 72.2 (SD = 13.3). CCS‐parents had lower resilience (mean = 69.5; SD = 12.4) than in comparison parents (mean = 73.9; SD = 13.6; *p* < 0.001). Among CCS‐parents, better general health was associated with higher resilience (*p* < 0.001), while living in French‐ or Italian‐speaking regions (vs. German‐speaking; *p* = 0.044) and higher levels of depression (*p* = 0.001) were associated with lower resilience.

**Conclusion:**

Our study supports the need to enhance resilience in parents of survivors, which may help to improve overall mental health and wellbeing of this population.

## 1 Introduction

A childhood cancer diagnosis impacts the psychological [1, 2], financial [3], and social situation of the family. Previous studies on parents of children diagnosed with cancer reported high stress and anxiety, poor health and wellbeing in parents and changes in family dynamics [4]. However, there has been limited investigation into the positive psychosocial adaptations, such as resilience [1]. Understanding resilience is crucial, as it is a valuable psychological resource for both survivors and their families, enabling them to effectively manage the long‐term challenges associated with cancer survivorship.

The American Psychological Association defines resilience as “the process of adapting well in the face of adversity, trauma, tragedy, threats or significant sources of stress” [5]. It is a product of an individual's appraisal of stress and the subsequent coping strategies used to manage it accordingly [6]. The relationship between stress and resilience is hypothesized to be non‐linear, following an inverted U‐shaped curve [7, 8]. Low levels of stress do not significantly challenge an individual and thus fail to foster resilience [7, 8]. Moderate stress promotes the development of coping mechanisms, enhancing resilience for future challenges [7, 8]. High or prolonged stress, conversely, may overwhelm adaptive systems, hindering resilience‐building and leading to negative outcomes such as anxiety, depression, somatization, emotional dysregulation, and burnout [7, 8, 9].

The experience of childhood cancer diagnosis could be a continuing source of stress for parents, considering the possible late effects of therapy, the persistent fear of secondary malignancies and late effects, or worries about the child's future [2, 10]. However, it remains unclear whether these prolonged challenges hinder or promote resilience over time.

Various factors contributing to resilience have been identified. Personal traits, coping mechanisms, social support networks, and access to mental health resources all enhance an individual's capacity to manage stressors and support mental wellbeing, thereby fostering resilience [11]. Conversely, risk factors such as chronic and repeated stress, lack of social support, and low socioeconomic status can hinder an individual's resilience and increase vulnerability to adverse mental health outcomes [12, 13]. A previous study including parents of leukemia survivors found that family support and self‐efficacy were significant factors in increasing parental resilience [14]. Yet, the single‐center study design and the exclusion of other cancer types limit the generalizability of the results.

Few studies have investigated the resilience in parents of childhood cancer survivors [14, 15, 16, 17]. These studies used different measurement tools, and some were exploratory without a comparison group [15, 17]. One study compared parents of survivors with parents of healthy children [14], and another study used bereaved parents as comparison [16]. None used a population‐based comparison group.

We aimed to explore the resilience of parents of childhood cancer survivors (CCS‐parents). We (a) described the resilience of CCS‐parents, parents from the general population (matched from the general population), and the Swiss general population (SGP), (b) compared the resilience of CCS‐parents and parents from the general population, and (c) identified sociodemographic and clinical characteristics, and physical and mental health outcomes associated with resilience among CCS‐parents. In this study, resilience was conceptualized as a dynamic process reflecting individuals' capacity to adapt to adversity, rather than a fixed trait.

## 2 Methods

This was a population‐based cross‐sectional study using data from the Swiss Childhood Cancer Survivor Study—Parents (SCCSS‐Parents).

### 2.1 Settings and Study Procedure

The Swiss Childhood Cancer Registry identified parents of childhood cancer survivors (CCS). Parents were eligible for the study if their child was (a) diagnosed with cancer at age ≤ 16 years, (b) Swiss resident at the time of diagnosis, (c) ≥ 5 years from diagnosis, and (d) ≥ 20 years old at study. Eligible parents were contacted by the former treating clinic. Study information was sent first, followed by two copies of the questionnaire (one for each parent) 2 weeks later. A first reminder was sent to non‐responders 4–6 weeks after the questionnaire, followed by a second reminder 4–6 weeks after the first. Data collection took place between January 2017 and February 2018.

For the parents from the general population, the Swiss Federal Statistical Office (SFSO) provided a representative sample of adults aged 18–75 years in 2015, stratified by age, sex, and language region (German, French, Italian). Study information was sent by the study team, followed by the questionnaire 2 weeks later. A reminder was sent to non‐responders 4–6 weeks after the questionnaire. The contact period was from May 2015 to June 2016. From this sample, we selected participants who had at least one child aged ≥ 20 years to match the age range of CCS in the CCS‐parents group.

All the study materials were available in German, French, and Italian, the major languages in Switzerland.

### 2.2 Measurements

#### 2.2.1 Primary Outcome

The Connor‐Davidson Resilience Scale (CD‐RISC) is a psychometric tool designed to assess an individual's level of resilience (including competence, 8 items; resistance, 7 items; positivity, 5 items; control, 3 items; and spirituality, 2 items) [18]. The scale consists of 25 self‐report items, each rated on a 5‐point Likert scale (from 0 “not true at all” to 4 “true nearly all the times”, minimum score 0, maximum score 100). We computed the resilience sum scores and the subscale mean scores. For missing responses, we imputed the mean of the responses to other items if < 25% (< 6 items) were missing.

#### 2.2.2 Sociodemographic Characteristics

The questionnaire included sex (male, female), age (41–65, > 65 years old), risk of poverty (CHF < 6000/month as household income for parent‐couples or CHF < 4500/month for single parents; based on Swiss Federal Statistical Office criteria [19] and as used in a previous publication [3]), education attainment (compulsory, vocational training, upper secondary, and university education), language region (German, French/Italian), employment status (unemployed, employed, retired), household size (1–2 people, 3 or more people), religious affiliation (any or none), and partnership status (with or without partner).

#### 2.2.3 Psychological and Physical Health Characteristics

We used the Brief Symptom Inventory (BSI 18) subscales for depression (6 items) and anxiety (6 items) [20, 21], validated in the Swiss population [22]. For each item, respondents expressed their distress during the previous 7 days on a 5‐point Likert scale (from 0 “not at all” to 4 “extremely”). Items of the two subscales were added to calculate the two sum scores. For participants missing one or two items per subscale, scale scores were calculated by summing all items, with missing values imputed using the rounded average of the remaining items. A T‐score was computed (with mean = 50, SD = 10) following the scoring manual [20].

General health was measured using the first item of the Short‐Form 36 version 2 (SF‐36v2) questionnaire “In general, would you say your health is?” with 5‐point Likert scale answers (from 1 “poor” to 5 “excellent”) [23]. The SF‐36 v2 has been validated in Switzerland. The item has been shown to be a valid measure for general health perception as used in other studies [23].

Partnership quality was measured through the “Relationship‐specific attachment scales for adults” (Beziehungsspezifische Bindungsskalen für Erwachsene) [24] containing 14 questions (from 1 “completely disagree” to 5 “completely agree”, possible range: 14–70). It has two subscales measuring attachment security (secure—anxious, 6 items) and perceived available support (dependent—independent, 8 items). Items that were phrased negatively were reverse‐coded. For < 20% missing values (≤ 2 items), the mean of the respective subscale was imputed. We used mean and standard deviation to summarize each subscale.

#### 2.2.4 Childhood Cancer Survivor‐Related Characteristics

CCS‐related characteristics of survivors were obtained from the Swiss Childhood Cancer Registry, namely: sex (male, female), age at study (< 30, 30–40, > 40 years old), age at diagnosis (< 5, 5–10, > 10 years), time since diagnosis (< 20, 20–24, 25–29, ≥ 30 years), cancer diagnosis (leukemia, lymphoma, central nervous system [CNS] tumor, others), cancer therapy (surgery only, chemotherapy [may have had surgery], radiotherapy [may have had surgery and/or chemotherapy], and hematopoietic stem cell transplantation (HSCT) [may have had surgery and/or chemotherapy and/or radiotherapy]), relapse (yes, no). In the survey, parents were asked whether the survivor currently experienced any late effects (parent‐reported late‐effects: yes, no).

### 2.3 Data Analysis

We described the sociodemographic and psychological characteristics among CCS‐parents, parents from the general population, and the SGP using means, standard deviations (SD) and percentages. Chi‐squared test and students' *t*‐test were used for group comparison.

#### 2.3.1 Aim 1

We described the resilience sum score, and resilience subscale mean scores using means and SD for each of the three groups.

#### 2.3.2 Aim 2

We used linear regression to compare the resilience among CCS‐parents and parents from the general population. To account for differences in sociodemographic and psychological characteristics between CCS‐parents and parents from the general population, we calculated the marginal means of the resilience sum score using a multivariable linear regression model, adjusting for sociodemographic and psychological characteristics that significantly differed between the two groups (with *p* < 0.05).

#### 2.3.3 Aim 3

We used multilevel linear regression models to determine sociodemographic, psychological and physical health, and CCS‐related characteristics associated with resilience in CCS‐parents. This analysis used a random intercept with parent‐couples (household) as clusters, accounting for the assessment of within‐household correlations. Resilience sum score was the dependent variable. Independent variables were sociodemographic (sex, age, risk of poverty, language region, education, employment, household size, religious affiliation, and partnership status), psychological and physical health (depression, anxiety, general health, and partnership quality), and CCS‐related (sex, age at study, age at diagnosis, time since diagnosis, cancer diagnosis, cancer therapy, parent‐reported late effects, and relapse) characteristics. Variables significant at *p* < 0.05 in the univariable regressions were included in the multivariable regression.

All analyses were conducted in Stata 18.5 (Statacorp, TX) using two‐tailed tests, with *p* < 0.05 was considered statistically significant.

### 2.4 Ethical Considerations

Ethical approval was granted through the Ethics Committee of Northwest and Central Switzerland (EKNZ 2015–075; 26 March 2015). Written informed consent was obtained from all individual participants included in the study.

## 3 Results

**FIGURE 1:**
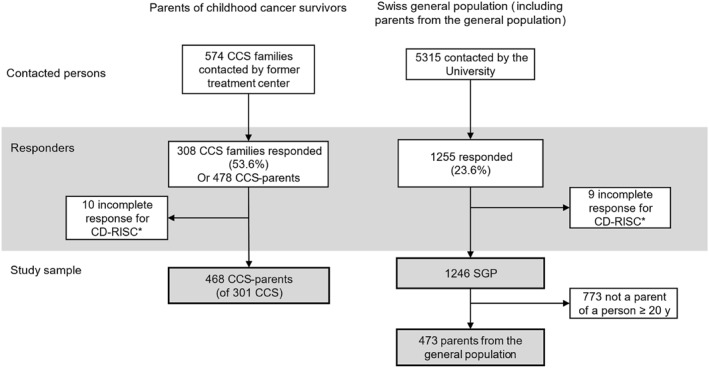
Enrollment of parents of childhood cancer survivors (CCS‐parents) and the Swiss general population (and the parents from the general population). *Responses with ≥ 25% of missing items were removed from the analysis as they did not meet the criteria for missing imputation of CD‐RISC. CCS, childhood cancer survivor; CD‐RISC, Connor‐Davidson Resilience Scale; SGP, Swiss general population; y, years.

Parents of 574 childhood cancer survivors were contacted by the former treatment centers, and 478 CCS‐parents of 301 survivors responded (response rate 53.6%) (Figure 1). We included 468 CCS‐parents with resilience data in the analysis (10 CCS‐parents removed for incomplete response). From the Swiss general population (SGP), 5644 adults were representative sampled, of which 5315 could be contacted. From those contacted, 1255 responded (response rate 23.6%), of which 1246 had valid responses on the CD‐RISC and were included in the analysis. From this sample, we identified 473 adults who had at least one child aged ≥ 20 years; these formed the parents from the general population.

CCS‐parents (58.5% females) had a mean age of 62.2 years (SD = 6.8, range 45–85 years; Table 1). Parents from the general population (57.5% females) had a mean age of 62.1 years (SD = 7.9, range 40–76 years). Participants from the Swiss general population (58.2% females) had a mean age of 48.9 years (SD = 15.3, range 18–74). Both CCS‐parents and parents from the general population were mostly > 65 years old (59.9% and 59.0%), employed (55.8% and 49.6%), completed vocational education (53.4% and 54.9%), and lived in the German‐speaking region of the country (73.3% and 71.0%) (Table 1). For the SGP, most participants reported no risk of poverty (72.2%), had completed vocational training (47.9%), lived in the German‐speaking region (73.0%), and were employed (69.3).

**TABLE 1:** Characteristics of parents of childhood cancer survivors (CCS‐parents), parents from the general population, and the Swiss general population (SGP).

	CCS‐parents	Parents from the general population^a^	SGP
*N*	468	473	1246
Sociodemographic characteristics			
Sex			
Male	194 (41.5%)	201 (42.5%)	521 (41.8%)
Female	274 (58.5%)	272 (57.5%)	725 (58.2%)
Age (in years), mean (SD)	62.2 (6.8%)	62.1 (7.9)	48.9 (15.3)
Age (in categories)			
18–25 years	—	—	105 (8.4%)
26–40 years	—	—	294 (23.6%)
41–65 years	186 (40.1%)	194 (41.0%)	647 (51.9%)
> 65 years	278 (59.9%)	279 (59.0%)	200 (16.1%)
Risk of poverty^b^			
At risk	129 (28.5%)	151 (32.8%)	336 (27.8%)
No risk	324 (71.5%)	310 (67.2%)	874 (72.2%)
Education			
Compulsory schooling	53 (12.4%)	40 (9.2%)	99 (8.4%)
Vocational training	229 (53.4%)	240 (54.9%)	562 (47.9%)
Upper secondary education	147 (34.3%)	157 (35.9%)	216 (18.3%)
University	80 (18.3%)	76 (17.7%)	296 (25.1%)
Language region	77 (17.6%)	71 (16.6%)	
German	343 (73.3%)	336 (71.0%)	909 (73.0%)
French or Italian	125 (26.7%)	137 (29.0%)	337 (27.0%)
Employment status			
Unemployed	38 (8.4%)	45 (9.8%)	149 (12.3%)
Employed	253 (55.8%)	228 (49.6%)	841 (69.3%)
Retired	162 (35.8%)	187 (40.7%)	224 (18.5%)
Household size			
1 or 2	310 (71.1%)	325 (74.4%)	694 (55.3%)
3 or more	126 (28.9%)	112 (25.6%)	561 (44.7%)
Religious affiliation			
Any	389 (83.1%)	352 (75.5%)	894 (72.4)
None	79 (16.9%)	114 (24.5%)	341 (27.6)
Partnership status			
With partner	407 (89.8%)	392 (85.0%)	945 (78.1%)
Without partner	46 (10.2%)	69 (15.0%)	265 (21.9%)
Psychological characteristics			
Psychological distress (BSI‐18)			
Anxiety, mean (SD)	49.25 (10.72)	48.29 (10.35)	49.94 (10.67)
Depression, mean (SD)	49.00 (9.88)	49.08 (9.14)	50.30 (10.0)
General health (SF‐36), mean (SD)^c^	2.37 (0.80)	2.52 (0.78)	2.38 (0.81)
Partnership quality			
Attachment security, mean (SD)	2.98 (0.31)	2.97 (0.34)	2.90 (0.71)
Perceived available support, mean (SD)	3.73 (0.49)	3.69 (0.52)	4.31 (0.65)
CCS‐related characteristics (*N* = 301)			
Sex			
Male	168 (55.8%)		
Female	133 (44.2%)		
Age at study			
< 30 years	125 (41.5%)		
30–40 years	134 (44.5%)		
> 40 years	42 (14.0%)		
Age at diagnosis			
< 5 years	110 (36.5%)		
5–10 years	86 (28.6%)		
> 10 years	105 (34.9%)		
Time since diagnosis			
< 20 years	85 (28.2%)		
20–24 years	71 (23.6%)		
25–29 years	77 (25.6%)		
≥ 30 years	68 (22.6%)		
Cancer diagnosis			
Leukemia	102 (33.9%)		
Lymphoma	54 (17.9%)		
CNS	36 (12.0%)		
Others	109 (36.2%)		
Cancer therapy			
Surgery	37 (12.3%)		
Chemotherapy	166 (55.3%)		
Radiotherapy	78 (26.0%)		
HSCT	19 (6.3%)		
Parent‐ reported late‐effects			
Yes	121 (41.7%)		
No	169 (58.3%)		
Relapse			
Yes	37 (12.3)		
No	264 (87.7)		

Abbreviations: BSI, Brief Symptom Inventory; CCS, childhood cancer survivor; CNS, central nervous system; HSCT, hematopoietic stem cell transplantation; SD, standard deviation; SF‐36, Short Form‐36; SGP, Swiss general population.

^a^ Parents from the general population are derived from the Swiss general population, which included parents with at least one child aged ≥ 20 years at study.

^b^ Risk of poverty is classified as household earning CHF < 4500/month for single parents, and CHF < 6000/month for parent‐couples (based on Swiss Federal Statistical Office report on poverty in Switzerland (Bundesamt für Statistik. Armut in der Schweiz: Konzepte, Resultate und Methode‐Ergebnisse auf der Basis von SILC 2008 bis 2010 [Poverty in Switzerland: concepts, results and methods—results based on SILC 2008–2010]. 2012) and previous study (Mader, et al. Pediatr Blood Cancer. 2017; 64(8))).

^c^ First item in Short Form 36 (SF‐36) was used “In general, would you say your health is: excellent, very good, good, fair, poor”.

Several sociodemographic and psychological characteristics differed between CCS‐parents and parents from the general population. There were more CCS‐parents with religious affiliation (83.1%), and in a partnership (89.8%) than parents from the general population (with religious affiliation: 75.5%; in a partnership: 85.0%; Table 1, Supporting Information S1: Appendix Table S1). CCS‐parents had lower general health (mean = 2.37, SD = 0.80) than parents from the general population (mean = 2.52, SD = 0.78, *p* = 0.005; Table 1, Supporting Information S1: Appendix Table S1). All these variables were adjusted in the regression models.

Among the 301 survivors, most were males (*n* = 168, 55.8%), younger than 40 years old at study (*n* = 259, 85.8%), and diagnosed in early childhood (< 5 years, *n* = 110, 36.5%), Leukemia was the most common diagnosis (*n* = 102, 33.9%), and more than half underwent chemotherapy (*n* = 1166, 55.3%) (Table 1).

### 3.1 Resilience of CCS‐Parents, Parents From the General Population and Swiss General Population (Aim 1)

**FIGURE 2:**
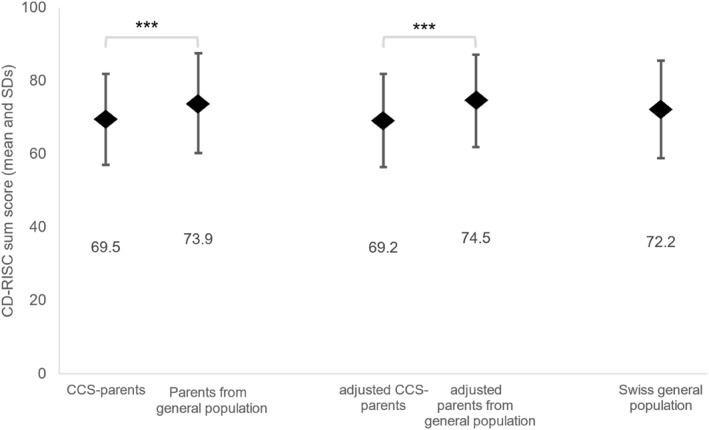
Resilience (CD‐RISC) sum scores of parents of childhood cancer survivors (CCS‐parents), parents from the general population, and the Swiss general population (SGP). Estimated marginal means adjusted by general health (SF‐36), religious affiliation and living in partnership which were statistically significant difference between CCS‐parents and parents from the general population (see Supporting Information S1: Appendix Table S1).***p value < 0.001; **p value < 0.05.

Resilience sum scores were 69.5 (SD = 12.4) for CCS‐parents, 73.9 (SD = 13.6) for parents from the general population, and 72.2 (SD = 13.3) for the Swiss general population (Figure 2). Results for resilience subscales (competence, resistance, positivity, control, spirituality) can be found in the Appendix (Supporting Information S1: Appendix Table S2). Positivity had the highest mean score, and spirituality had the lowest mean among the five subscales. This finding is consistent across all three groups (CCS‐parents, parents from the general population, and SGP) (Supporting Information S1: Appendix Table S2).

### 3.2 Resilience Comparison Between CCS‐Parents and Parents From the General Population (Aim 2)

Resilience was lower for CCS‐parents compared to parents from the general population (*p* < 0.001) and the SGP (*p* = 0.001) (Figure 2; Supporting Information S1: Appendix Table S2). After adjusting estimates for religious affiliation, partnership status, and general health, resilience remained lower for CCS‐parents compared to parents from the general population (CCS‐parents: *M* = 69.2, SD = 12.2; parents from the general population: *M* = 74.5, SD = 12.6, *p* < 0.001) (Figure 2).

### 3.3 Factors Associated With Resilience Among CCS‐Parents (Aim 3)

In univariable models, education, and language region were significantly associated with resilience (Table 2). CCS‐parents with university education reported higher resilience compared to those with only compulsory education (*B* = 4.41, 95% CI 0.03 to 8.78, *p* = 0.048). Resilience was lower among CCS‐parents residing in French/Italian‐speaking regions compared to those in the German‐speaking region (*B* = −4.63, 95% CI −7.15 to −2.12, *p* < 0.001).

**TABLE 2:** Determinants of resilience in parents of childhood cancer survivors (CCS‐parents) using multilevel linear regression.

	Univariable analysis			Multivariable analysis		
	Coefficient	95% CI	*p*‐value	Coefficient	95% CI	*p*‐value
Sociodemographic characteristics						
Sex (ref: Male)						
Female	0.77	(−1.52, 3.06)	0.510			
Age in categories (ref: ≤ 65 years)						
Older age (> 65 years)	−1.99	(−4.30, 0.32)	0.091			
Risk of poverty (ref: no risk)^a^						
At risk	−2.31	(−4.89, 0.27)	0.079			
Education (ref: Compulsory schooling)						
Vocational training	1.95	(−1.72, 5.61)	1.95	0.01	(−3.62, 3.65)	0.994
Upper secondary education	3.75	(−0.55, 8.06)	0.088	1.88	(−2.29, 6.05)	0.377
University	**4.41**	**(0.03, 8.78)**	**0.048**	1.19	(−3.02, 5.40)	0.580
Language region (ref: German)						
French or Italian	**−4.63**	**(−7.15, −2.12)**	**< 0.001**	**−2.73**	**(−5.38, −0.08)**	**0.044**
Employment status (ref: Unemployed)						
Employed	2.86	(−1.35, 7.07)	0.183			
Retired	1.44	(−2.91, 5.80)	0.516			
Household size (ref: 1–2 persons)						
3 or more	1.27	(−1.27, 3.81)	0.326			
Religious affiliation (ref: Any)						
None	0.79	(−2.22, 3.80)	0.606			
Partnership status (ref: Without partner)						
With partner	0.77	(−3.04, 4.58)	0.690			
Psychological characteristics						
Psychological distress (BSI 18)^b^						
Anxiety	**−0.36**	**(−0.47, −0.26)**	**< 0.001**	−0.09	(−0.24, 0.04)	0.207
Depression	**−0.42**	**(−0.53, −0.31)**	**< 0.001**	**−0.25**	**(−0.41, −0.10)**	**0.001**
General health (SF‐36)^b^	**5.62**	**(0.26, 0.47)**	**< 0.001**	**4.24**	**(2.71, 5.76)**	**< 0.001**
Partnership quality^b^						
Attachment security	**4.07**	**(2.22, 5.91)**	**0.001**	0.18	(−0.03, 0.39)	0.092
Perceived available support	−1.34	(−3.01, 0.32)	0.114			
CCS‐related characteristics						
Gender (ref: male)						
Female	1.11	(−1.16, 3.38)	0.336			
Age at study (ref: < 30 years)						
30–40 years	−0.69	(−3.10, 1.73)	0.575			
> 40 years	0.43	(−3.18, 4.04)	0.816			
Age at diagnosis (ref: < 5 years)						
5–10 years	**−2.83**	**(−5.59, −0.06)**	**0.045**	−1.44	(−4.18, 1.31)	0.304
> 10 years	−1.11	(−3.79, 1.57)	0.417	0.53	(−2.18, 3.25)	0.698
Time since diagnosis (ref: < 20 years)						
20–24 years	0.81	(−2.30, 3.92)	0.611			
25–29 years	1.42	(−1.67, 4.50)	0.367			
≥ 30 years	−0.79	(−4.05, 2.47)	0.635			
Cancer diagnosis (ref: Leukemia)						
Lymphoma	−0.27	(−3.57, 3.03)	0.874			
CNS	−2.30	(−6.12, 1.51)	0.237			
Others	0.24	(−2.45, 2.93)	0.861			
Cancer therapy (ref: Chemotherapy)						
Surgery	**−4.25**	**(−7.73, −0.77)**	**0.017**	−2.77	(−6.32, 0.77)	0.069
Radiotherapy	−0.70	(−3.38, 1.96)	0.603	0.86	(−1.94, 3.66)	0.546
HSCT	3.10	(−1.89, 8.10)	0.223	**6.29**	**(1.27, 11.31)**	**0.014**
Parent‐reported late‐effects (ref: no)						
Yes	0.69	(−1.56, 2.94)	0.546			
Relapse (ref: no)						
Yes	−0.79	(−4.16, 2.58)	0.645			

*Note: p* values < 0.05 are indicated in bold font.

Abbreviations: BSI 18, Brief Symptom Inventory; CCS, childhood cancer survivor; CI, confidence interval; CNS, central nervous system; HSCT, hematopoietic stem cell transplantation; SF‐36, Short Form 36.

^a^ Risk of poverty is classified as household earning CHF < 4500/month for single parents, and CHF < 6000/month for those parent couple based on Swiss Federal Statistical Office report on poverty in Switzerland and previous study (Mader, et al. Pediatr Blood Cancer. 2017; 64(8) and Bundesamt für Statistik. Armut in der Schweiz: Konzepte, Resultate und Methode‐Ergebnisse auf der Basis von SILC 2008 bis 2010 [Poverty in Switzerland: concepts, results and methods]. 2012).

^b^ These scores were fitted as continuous variables. As such, coefficients refer to incremental change in resilience per change in independent/predictor variable (BSI 18, SF‐36, or Partnership quality, namely attachment security and perceived available support).

For psychological characteristics, resilience was negatively associated with anxiety (*B* = −0.36, 95% CI −0.47 to −0.26, *p* < 0.001) and depression (*B* = −0.42, 95% CI −0.53 to −0.31, *p* < 0.001). Higher resilience was observed among those with better general health (*B* = 5.62, 95% CI 0.26 to 10.98, *p* = 0.040) and higher partnership quality (attachment security; *B* = 4.07, 95% CI 2.22 to 5.91, *p* < 0.001).

Among CCS‐related characteristics, a cancer diagnosis at ages 5–10 years was associated with lower resilience (*B* = −2.83, 95% CI −5.59 to −0.06, *p* = 0.045) compared to those diagnosed at < 5 years old. Surgery only was also associated with lower resilience (*B* = −4.25, 95% CI −7.73 to −0.77, *p* = 0.017) compared to chemotherapy.

In the multivariable model, residence in a French/Italian‐speaking region (*B* = −2.73, 95% CI −5.38 to −0.08, *p* = 0.044) and with higher levels of depression (*B* = −0.25, 95% CI −0.41 to −0.10, *p* = 0.001) were associated with lower resilience. Better general health (*B* = 4.24, 95% CI 2.71 to 5.76, *p* < 0.001) and a history of HSCT compared to chemotherapy (*B* = 6.29, 95% CI 1.27 to 11.31, *p* = 0.014) were associated with higher resilience.

## 4 Discussion

Parents of childhood cancer survivors were found to have lower resilience compared to parents in the general population. Moreover, we identified characteristics associated with resilience. Parents living in the French‐ and Italian‐speaking regions of Switzerland, and those with higher levels of depression showed lower resilience. Whereas parents who reported better general health had higher resilience.

Families often face immense stress upon a diagnosis of cancer in their child, which continues during therapy. After “successfully” navigating such challenges, one may expect that this experience would lead to high resilience for parents of survivors. This is compatible with the literature on stress‐resilience theory [7, 8, 9], which hypothesized that previous difficult situations could result in higher resilience that would be helpful in subsequent exposure to stressful events. However, our findings showed that parents of survivors had lower resilience, confirming findings from previous studies among 57 parents of acute lymphocytic leukemia survivors in Norway [14], and a single center study among 96 parents in the US [25].

The lower resilience observed in parents of survivors likely reflects multiple contributing factors, including sociodemographic, psychological, and survivorship‐related characteristics [14, 16, 26]. A plausible explanation is that the continuous or long‐term exposure to stress may prevent resilience‐building [6, 7, 8]. Our previous work showed that half of the parents of survivors reported ongoing concerns, the most common of which were worries about late‐effects (33%) and relapse or secondary malignancy (28%) [2, 10]. Additional studies using the same dataset revealed significant economic disruptions, including job loss or changes and reduced income, which can place further strain on the family unit [3]. Around one‐fifth of parents also expressed adverse outcomes related to their mental health, altered family dynamics, and reduced social support networks [10]. These ongoing pressures might limit both internal and external resources and contribute to lower resilience, with lasting effects well into the survivorship period.

Differences in resilience across language regions may reflect underlying social, cultural, and contextual factors not captured in our survey. Higher resilience in the German‐speaking population has also been reported in another study using the same scale [27]. e.g., family values in the German‐speaking part of Switzerland tend to emphasize independence and autonomy, whereas values in the French‐ and Italian‐speaking parts emphasize emotional and psychological interdependence [28]. Both independence and autonomy are associated with competence (i.e., the belief in one's abilities and skills to deal with challenges effectively) and control (i.e., sense of agency and influence over one's outcomes and responses to adversity), both of which may contribute to the development and experience of resilience. Another possible reason is a cultural variation of resilience as a construct [29], that is different cultures may have varying understanding of resilience [30]. In the German culture, the emphasis on individuality may align more closely with the CD‐RISC's constructs, whereas in the French culture, social connectedness is considered a key resource, which may not be adequately captured by the resilience scale [31].

Our analysis also demonstrated that higher depression levels were associated with lower resilience. Individuals with lower resilience tend to have lower ability to cope with stress and challenges in life [32]. As such, they are more likely to suffer psychological distress, including depression [32]. A systematic review found a higher psychological distress burden in parents of survivors compared to the general population [33]. Our findings add that the high psychological distress in parents of survivors may be partly explained by their lower resilience. Moreover, we also found a positive association of general health with resilience. Components of resilience, such as positivity (having a positive attitude to change) or control (perceived control over outcome) are both related to physiological and psychological health [34], thus, highlighting the strong association of physical and mental health with resilience [34].

Past intensive therapy was also associated with resilience. Previous HSCT was associated with higher resilience levels in multivariable analysis. Parents of survivors who previously underwent HSCT might experience higher competence, self‐efficacy, and confidence after having successfully finished a particularly difficult and life‐threatening therapy, which could result in higher resilience. Higher resilience may also be related to higher post‐traumatic growth after facing higher risk of mortality during HSCT, as shown in our previous study [35]. However, due to the low number of parents whose children underwent HSCT, this result needs to be interpreted with caution.

### 4.1 Strengths and Limitations

Our study was based on a population‐based sampling strategy. The Swiss Childhood Cancer Registry systematically identified CCS, which were used to identify parents of survivors across all Swiss clinics treating children for childhood cancer. The parents from the general population came from a representative sample of the Swiss general population. Thus, our study design is more methodologically robust, compared to most previous studies that used single‐center data [14, 26] and no population‐based comparisons [14, 26]. Moreover, we employed a clustered modeling approach, treating parent‐couples as a unit of analysis. This method accounts for intra‐family correlations and offers a more family‐centered perspective compared to traditional, non‐clustered analytical approaches. Finally, this is one of the few quantitative studies on family resilience in cancer survivorship, one of the larger studies on parents of survivors, and one that has a higher proportion of fathers making it more gender‐role representative compared to other studies.

However, our study has several limitations that warrant careful consideration. First, our use of cross‐sectional analysis provides only a snapshot in time, preventing us from establishing causality between sociodemographic, psychological, and survivor‐related factors and resilience. Reverse causality remains a plausible explanation for our findings. Second, our reliance on a single time point measurement of resilience fails to capture its dynamic nature. Although some experts argue that resilience is static and deeply rooted in personality traits [36], this viewpoint contrasts with the evolving understanding of resilience as a dynamic construct from the previous literature [7, 8, 9]. Therefore, measuring resilience at multiple time points through longitudinal assessments would better reflect its dynamic and changing nature. Third, other sociodemographic factors, that could affect resilience, were not collected, which include tight family relationships, presence and quality of a social network, and participation in parental support groups, as they comprise crucial resource for resilience building [16, 25, 26]. Finally, although the models were adjusted for relevant covariates, the difference in response rates between the two groups may indicate selection bias and could partly explain the observed differences. Finally, although the CD‐RISC is widely used, its content has not been specifically validated for the Swiss population.

### 4.2 Clinical Implications and Outlook

Lower resilience among parents of childhood cancer survivors may reflect ongoing stress or lasting effects of the past experience. Nevertheless, fostering resilience among this group is important. First, it is hypothesized to have an impact on the development of psychological distress, emotional dysregulation, relationship problems, or burnout among parents [37]. Investigating resilience, and its determinants, is critical to predict and identify who will develop poor mental health [37]. Second, resilience may have long‐term effects on family dynamics [12, 38]. Childhood cancer not only disrupts the life of the affected child but may also have lasting effects on the parenting of healthy siblings and partner relationships among parents [38]. Studying resilience in parents can provide insights into how families adapt, cope, and maintain cohesion in the face of adversity. Identifying resilient behaviors can help develop family‐centered interventions that enhance overall family functioning. Third, the resilience of parents may have a direct impact on survivors' health and wellbeing. Parental support and coping strategies during times of stress and adversities play a vital role in creating a positive and supportive environment for the child's long‐term health [39]. Thus, understanding resilience factors in parents can contribute to interventions that optimize survivor outcomes by fostering a nurturing and supportive family environment. Future studies should venture into the application of resilience‐based programs in supporting families of survivors. Key components of these resilience‐based programs include stress management, goal‐setting, cognitive reframing, and meaning making, all have been proven to improve the resilience of parents whose child was recently diagnosed of cancer [40].

## 5 Conclusion

Our study revealed that parents of childhood cancer survivors report lower resilience compared to parents and individuals from the general population. Parents living in the French‐ and Italian‐speaking regions of Switzerland and those with higher depression levels had lower resilience. In contrast, higher resilience was observed in parents who reported better general health. Strengthening resilience may help families navigate the long‐term challenges of childhood cancer survivorship, ultimately fostering improved mental health, family cohesion, and better outcomes for survivors.

## Funding

The Swiss Childhood Cancer Survivor Study—Parents was funded by the Swiss National Science Foundation (Grant No. 100019_153268/1; and 10001C_182129/1) and the Kinderkrebshilfe Schweiz.

## Ethics Statement

Ethical approval was granted through the Ethics Committee of Northwest and Central Switzerland (EKNZ 2015–075; 26 March 2015). Written informed consent was obtained from all individual participants included in the study.

## Conflicts of Interest

The authors declare no conflicts of interest.

## Supporting information


Supporting Information S1

## Data Availability

The data that support the findings of this study are available from the corresponding author upon reasonable request.
