# Supplementary material for: Resilience in Parents of Childhood Cancer Survivors: Results From the Swiss Childhood Cancer Survivors Study—Parents
Source: Psychooncology. 2026 Mar 30;35(4):e70443. doi: 10.1002/pon.70443 (PMC13036291; doi:10.1002/pon.70443)
Supplement: Supplementary file 1 — Supporting Information S1 [file PON-35-e70443-s001.docx]

STROBE Statement—checklist of items that should be included in reports of observational studies

|  | Item No. | Recommendation | Line  No. |
| --- | --- | --- | --- |
| **Title and abstract** | 1 | (*a*) Indicate the study’s design with a commonly used term in the title or the abstract | L69 |
|  |  | (*b*) Provide in the abstract an informative and balanced summary of what was done and what was found | L68-83 |
| Introduction | | | |
| Background/rationale | 2 | Explain the scientific background and rationale for the investigation being reported | L93-127 |
| Objectives | 3 | State specific objectives, including any prespecified hypotheses | L129-133 |
| Methods | | | |
| Study design | 4 | Present key elements of study design early in the paper | L136-137 |
| Setting | 5 | Describe the setting, locations, and relevant dates, including periods of recruitment, exposure, follow-up, and data collection | L140-155, |
| Participants | 6 | (*a*) *Cross-sectional study*—Give the eligibility criteria, and the sources and methods of selection of participants | L140-143, L148-150 |
| Variables | 7 | Clearly define all outcomes, exposures, predictors, potential confounders, and effect modifiers. Give diagnostic criteria, if applicable | L158-205 |
| Data sources/ measurement | 8* | For each variable of interest, give sources of data and details of methods of assessment (measurement). Describe comparability of assessment methods if there is more than one group | L158-205 |
| Bias | 9 | Describe any efforts to address potential sources of bias | L215-218 |
| Study size | 10 | Explain how the study size was arrived at | n.a. (included all participants) |
| Quantitative variables | 11 | Explain how quantitative variables were handled in the analyses. If applicable, describe which groupings were chosen and why | L211-212  L214-218  L220-229 |
| Statistical methods | 12 | (*a*) Describe all statistical methods, including those used to control for confounding | L211-212  L214-218  L220-229 |
|  |  | (*b*) Describe any methods used to examine subgroups and interactions | n.a. |
|  |  | (*c*) Explain how missing data were addressed | L165-166  L180-183  L194-196 |
|  |  | (*d*) *Cross-sectional study*—If applicable, describe analytical methods taking account of sampling strategy | L221 |
|  |  | (*e*) Describe any sensitivity analyses | n.a. |
| **Results** |  |  |  |
| Participants | 13* | (a) Report numbers of individuals at each stage of study—eg numbers potentially eligible, examined for eligibility, confirmed eligible, included in the study, completing follow-up, and analysed | L240-245 |
|  |  | (b) Give reasons for non-participation at each stage | n.a. |
|  |  | (c) Consider use of a flow diagram | Figure 1 |
| Descriptive data | 14* | (a) Give characteristics of study participants (eg demographic, clinical, social) and information on exposures and potential confounders | Table 1 |
|  |  | (b) Indicate number of participants with missing data for each variable of interest | n.a. |
| Outcome data | 15* | *Cross-sectional study—*Report numbers of outcome events or summary measures | Table S2 |
| Main results | 16 | (*a*) Give unadjusted estimates and, if applicable, confounder-adjusted estimates and their precision (eg, 95% confidence interval). Make clear which confounders were adjusted for and why they were included | Figure 2  Table 2  Table S2 |
|  |  | (*b*) Report category boundaries when continuous variables were categorized | Figure 2 |
|  |  | (*c*) If relevant, consider translating estimates of relative risk into absolute risk for a meaningful time period | n.a. |

| Other analyses | 17 | Report other analyses done—eg analyses of subgroups and interactions, and sensitivity analyses | n.a. |
| --- | --- | --- | --- |
| **Discussion** |  |  |  |
| Key results | 18 | Summarise key results with reference to study objectives | L303-306 |
| Limitations | 19 | Discuss limitations of the study, taking into account sources of potential bias or imprecision. Discuss both direction and magnitude of any potential bias | L365-381 |
| Interpretation | 20 | Give a cautious overall interpretation of results considering objectives, limitations, multiplicity of analyses, results from similar studies, and other relevant evidence | L447-448 |
| Generalisability | 21 | Discuss the generalisability (external validity) of the study results | L375-379 |
| Other information | |  |  |
| Funding | 22 | Give the source of funding and the role of the funders for the present study and, if applicable, for the original study on which the present article is based | L31-34 |

*Give information separately for cases and controls in case-control studies and, if applicable, for exposed and unexposed groups in cohort and cross-sectional studies.

**Note:** An Explanation and Elaboration article discusses each checklist item and gives methodological background and published examples of transparent reporting. The STROBE checklist is best used in conjunction with this article (freely available on the Web sites of PLoS Medicine at http://www.plosmedicine.org/, Annals of Internal Medicine at http://www.annals.org/, and Epidemiology at http://www.epidem.com/). Information on the STROBE Initiative is available at [www.strobe-statement.org](http://www.strobe-statement.org).

**Appendix Table S1.** Comparison of sociodemographic and psychological characteristics of parents of childhood cancer survivors (CCS-parents) and parents from general population*

|  | CCS-parents | Parents from general population^1^ | p value |
| --- | --- | --- | --- |
| N | 468 | 473 |  |
| Sociodemographic characteristics | | | |
| Sex |  |  |  |
| Male | 194 (41.5%) | 201 (42.5%) | 0.746 |
| Female | 274 (58.5%) | 272 (57.5%) |  |
| Age (in years), mean (SD) | 62.2 (6.8) | 62.1 (7.9) | 0.705 |
| Age (in categories) |  |  |  |
| ≤65 years | 186 (40.1%) | 194 (41.0%) | 0.772 |
| >65 years | 278 (59.9%) | 279 (59.0%) |  |
| Risk of poverty^2^ |  |  |  |
| At risk | 129 (28.5%) | 151 (32.8%) | 0.161 |
| No risk | 324 (71.5%) | 310 (67.2%) |  |
| Education |  |  |  |
| Compulsory schooling | 40 (9.2) | 53 (12.4) | 0.504 |
| Vocational training | 240 (54.9) | 229 (53.4) |  |
| Upper secondary education | 80 (18.3) | 76 (17.7) |  |
| University | 77 (17.6) | 71 (16.5) |  |
| Language Region |  |  |  |
| German | 343 (73.3%) | 336 (71.0%) | 0.440 |
| French or Italian | 125 (26.7%) | 137 (29.0%) |  |
| Employment status |  |  |  |
| Unemployed | 38 (8.4%) | 45 (9.8%) | 0.163 |
| Employed | 253 (55.8%) | 228 (49.6%) |  |
| Retired | 162 (35.8%) | 187 (40.7%) |  |
| Household size |  |  |  |
| 1 or 2 | 310 (71.1%) | 325 (74.4%) | 0.278 |
| 3 or more | 126 (28.9%) | 112 (25.6%) |  |
| Religious affiliation |  |  |  |
| Any | 389 (83.1%) | 352 (75.5%) | **0.004*** |
| None | 79 (16.9%) | 114 (24.5%) |  |
| Partnership |  |  |  |
| With partner | 407 (89.8%) | 392 (85.0%) | **0.028*** |
| Without partner | 46 (10.2%) | 69 (15.0%) |  |
| Psychological characteristics | | | |
| Psychological distress (BSI 18) |  |  |  |
| Anxiety, mean (SD) | 49.25 (10.72) | 48.29 (10.35) | 0.170 |
| Depression, mean (SD) | 49.00 (9.88) | 49.08 (9.14) | 0.899 |
| General health (SF-36), mean (SD)^3^ | 2.37 (0.80) | 2.52 (0.78) | **0.005*** |
| Partnership quality |  |  |  |
| Attachment security, mean (SD) | 2.98 (0.31) | 2.974 (0.34) | 0.470 |
| Relationship perceived available support, mean (SD) | 3.73 (0.49) | 3.69 (0.52) | **0.006*^2^** |

* Sociodemographic and psychological variables with significant group differences were included as covariates to adjust marginal means in the resilience sum score difference between CCS-parents and parents from general population.

Abbreviations, BSI, Brief Symptom Inventory; CCS, childhood cancer survivor; SD, standard deviation; SF-36, Short Form-36

^1^Parents from general population are derived from the Swiss general population and included persons with at least one child aged ≥20 years at study.

^2^Risk of poverty is classified as those earning CHF <4500/month for single parent, and CHF <6000/month for those parent-couple based on Swiss Federal Statistical Office report on poverty in Switzerland and previous study (Bundesamt für Statistik. Armut in der Schweiz: Konzepte, Resultate und Methode-Ergebnisse auf der Basis von SILC 2008 bis 2010 [Poverty in Switzerland: concepts, results and methods]. 2012.; and Mader L., et al, 2019. Pediatr Blood Cancer, 64(8))

^3^First item in Short Form 36 (SF-36) was used “In general, would you say your health is: excellent, very good, good, fair, poor”

**Appendix Table S2.** Resilience (CDRISC) sum score and subscale mean score across different groups*

| Resilience (number of items, possible score range) | CCS-parents  N=468 | Parents from general population  N=473 | Swiss general population  N=1,246 | |
| --- | --- | --- | --- | --- |
| Resilience sum score  (25 items, 0-125) | 69.5 (12.4) | 73.9 (13.6) | 72.2 (13.3) | |
| Subscale mean score | | | |  |
| Competence (8 items, 0-4) | 2.92 (0.59) | 3.04 (0.62) | 3.01 (0.62) | |
| Resistance (7 items, 0-4) | 2.96 (0.69) | 3.14 (0.71) | 3.03 (0.74) | |
| Positivity (5 items, 0-4) | 3.02 (0.57) | 3.27 (0.58) | 3.23 (0.57) | |
| Control (3 items, 0-4) | 2.56 (0.59) | 2.75 (0.64) | 2.68 (0.63) | |
| Spirituality (2 items, 0-4) | 2.13 (1.05) | 2.25 (1.10) | 2.06 (1.08) | |

*Abbreviations: CCS, childhood cancer survivor; SD, standard deviation*

^*^Based on Connor-Davidson Resilience Scale (CD-RISC), values expressed in mean and SDs.

Briefly, the definitions of CD-RISC subscales are as follows:

1. Competence was defined as belief in one’s ability, having high standards, and tenacity.
2. Resistance corresponds to trust in one’s instincts, tolerance of negative affect, and strengthening effects of stress.
3. Positivity was a construct that relates to the positive acceptance of change, and secure relationships.
4. Control was described as a sense influence over one’s outcomes and responses to adversity, and
5. Spirituality refers to the influences of spiritual belief or practices in providing strength and purpose.
